# Grape Pomace as a Replacement for Soybean Hulls in Corn Silage-Based Diets for Dairy Cows

**DOI:** 10.3390/vetsci13010087

**Published:** 2026-01-15

**Authors:** António J. M. Fonseca, Ana R. J. Cabrita

**Affiliations:** REQUIMTE, LAQV, ICBAS, School of Medicine and Biomedical Sciences, University of Porto, Rua Jorge Viterbo Ferreira 228, 4050-313 Porto, Portugal

**Keywords:** corn silage-based diets, dairy cows, grape pomace, protein degradation, soybean hulls

## Abstract

This study evaluated the effects of replacing soybean hulls with dried grape pomace (DGP) on feed intake, milk production and composition, and rumen N degradability in dairy cows. A 3 × 3 Latin square design was used with three rumen-fistulated Holstein cows, three corn silage-based diets containing 0%, 3%, or 6% of DGP, and three 23-day periods. Measurements were taken from days 15 to 21, with in situ incubations of two protein sources during the last 2 days. At trial end, cows continued on experimental diets to determine the degradability of soybean hulls and DGP. Compared to soybean hulls, DGP presented higher fiber content and lower in situ rumen dry matter and N degradability. Condensed tannin disappearance from DGP increased over time, reaching almost 50% after 48 h. Diet did not affect feed intake, milk production, and composition, except for milk urea N and ruminal total short-chain fatty acids concentration, which showed a quadratic response, suggesting both imbalances between fermentable energy and rumen degradable protein and potential effects of DGP bioactive compounds on rumen function. The N degradation rate of rapeseed meal was unaffected, but soybean meal degradability tended to increase with higher DGP inclusion. This was also detected when combining protein sources across diets. Although DGP influenced rumen function, further research integrating omics and detailed microbiota profiling is needed. Overall, despite its low energy content and rumen degradability, DGP comprises a sustainable feed resource for high-producing animals, supporting circular economy approaches and mitigating the environmental impacts of grape pomace disposal.

## Simple Summary

The valorization of dried grape pomace (DGP), a by-product of the wine industry, in ruminant feeding supports circular economy principles, reduces reliance on imported feedstuffs, and mitigates environmental issues related to grape pomace disposal. A 3 × 3 Latin square design was conducted using three rumen-fistulated Holstein cows fed corn silage-based diets containing 0%, 3%, or 6% DGP, replacing soybean hulls, over three 23-day periods. In situ degradability of DGP, soybean hulls, and two protein sources was assessed. Compared with soybean hulls, DGP exhibited higher fiber content and lower ruminal dry matter and N degradability, with condensed tannin disappearance approaching 50% after 48 h. Dietary treatment did not affect feed intake, milk yield, or composition, except for milk urea N and ruminal short-chain fatty acids, which showed quadratic responses, suggesting effects on rumen fermentation. Rapeseed meal degradability was unaffected, whereas soybean meal degradability tended to increase with increasing DGP inclusion. Overall, despite its low energy value and ruminal degradability, DGP represents a sustainable feed resource for dairy production.

## 1. Introduction

The European Union’s compound feed industry depends on imported raw materials from third-world countries, especially for high-protein feeds [1]. Portugal’s situation is particularly concerning, as it has very low self-sufficiency rates for cereals (18%, excluding rice), grain legumes (15%), and oilseeds, ca. 5% for sunflower and rapeseed [2]. In this context, it is urgent to promote and implement the “circular feed concept,” especially for ruminants, which are naturally efficient at using the “low-grade, non-human edible by-products” that emerge from the circular economy [3]. Like other wine-producing countries and regions, Portugal generates large quantities of grape pomace (GP) annually, 170,000 t in 2024, considering that it represents 25% of the waste generated by the wine industry [4]. Grape pomace is the main solid by-product obtained through the pressing and fermentation phases of wine production, comprising grape skins, pulp, and seeds remaining after winemaking, and it is either discarded as waste or utilized for wine alcohol production, fertilizer, or animal feed [5]. When dehydrated, it constitutes a traditional raw material used by the compound feed industry.

However, due to low energy value and high condensed tannin content, dehydrated GP (DGP) is typically used only to produce compound feed for rabbits and ruminants with low nutritional requirements. In rabbit feed, a low incorporation level of less than 5% [6] is commonly used as a source of indigestible functional fiber to improve intestinal health and reduce digestive disorders [7,8]. In ruminants, DGP is primarily used in compound feeds for heifers, beef cows, and sheep at levels up to 5–6% [6]. In addition to assumed nutritional restrictions, higher incorporation levels can darken the feed and alter its aroma, which are commercially undesirable. Moreover, DGP is traditionally used to formulate large-diameter pellets for use as grazing animal supplements during feed scarcity periods (e.g., summer and early fall, or after large fires) or throughout the year in severe drought scenarios, with high incorporation levels ranging from 10% to 20%. Due to its nutritional characteristics, most dairy and feedlot nutritionists consider DGP a non-raw material, not considering its use when formulating diets. Thus, the amount of DGP used by the industry is reduced, falling far below the desired level from a circular economy perspective.

Nevertheless, GP is rich in bioactive and antimicrobial compounds, and is proving to be a promising antimicrobial alternative in feed, as critically reviewed by Hassan and Kosir [9]. Recent in vitro and in vivo studies have observed shifts in rumen function with GP supplementation up to 20% dry matter (DM) basis, namely in the fibrolytic population and ammonia-N concentration [10,11,12]. Moreover, Kara and Öztaş [13] demonstrated in vitro that fermented GP containing fermentative yeast and bacteria had an anti-methanogenic effect without compromising energy availability or fermentation efficiency. Notably, Akter and Li [14] reported that feeding dairy cows fresh GP at 10% and 15% DM reduced enteric methane emissions. The content of phenolic compounds and organic acids may have contributed to this reduction. In vivo studies with beef cattle have also shown that GP can increase antioxidant enzyme activity, particularly catalase, as well as total antioxidant capacity [11], and improve immunity [12] without affecting performance. Overall, these findings indicate that GP constitutes a sustainable feed resource that not only promotes animal health but also contributes to strategies aimed at reducing the environmental footprint of high-performance ruminants. Furthermore, the valorization of grape and winery by-products as animal feed mitigates the detrimental effects of their disposal, such as contamination of ground and surface waters, attraction of disease-spreading vectors, and the depletion of oxygen in soil and groundwater, which can ultimately affect wildlife [15].

Therefore, the objective of this project was to evaluate the effects of replacing an energy fiber source (soybean hulls; SH) with DGP in a common complementary compound feed formula for corn silage-based TMR on the voluntary intake, milk production, and composition of dairy cows. Although DGP and SH differ considerably in chemical composition, their similar crude protein (CP) and neutral detergent fiber (NDF) contents allow direct replacement while keeping these dietary components relatively stable. Direct substitution alters the balance of other dietary nutrients, particularly the ratio between fermentable metabolizable energy and rumen-degradable protein (RDP) contents, but has the advantage of avoiding the confounding effects that arise when multiple raw materials are changed in the compound feed. Considering the putative shifts in rumen function, we anticipated that DGP could impact rumen protein degradability. To test this hypothesis, we determined the in situ degradability of two common protein sources with known different degradation rates (rapeseed meal, RSM; and soybean meal, SBM) in cows fed different amounts of DGP.

## 2. Materials and Methods

The experiment was carried out at the Vairão Agricultural Campus of the School of Medicine and Biomedical Sciences, University of Porto (ICBAS-UP; Vila do Conde, Portugal), and all procedures involving animals were approved by the Animal Ethics Committee of the ICBAS-UP and licensed by the Portuguese General Directorate of Food and Veterinary Affairs (permit #0421/000/000/2021).

### 2.1. Animals, Experimental Design, Treatments, and Management

Three third-lactation Holstein cows, averaging 102 days in milk (SD = 24.0) and 43 kg/d of milk (SD = 3.6), were used in the experiment, which was carried out between June and August 2024. Each cow was fitted with a 10 cm-diameter rumen cannula (Bar Diamond Inc., Parma, ID, USA). The cows were housed in adjacent individual boxes ranging from 10 to 11.5 m^2^, which encouraged contact, grooming, and socializing. The cows had continuous access to drinking water and mineral salt blocks. The cows had access to an outdoor paddock from 10:00 a.m. to 12:30 p.m.

The animals were randomly assigned to dietary treatment sequences in a 3 × 3 Latin square design. Each experimental period lasted for 23 d, with measurements performed from d 15 to 21, and in situ incubations performed during the last 2 d. Experimental diets contained (DM basis) 53% corn silage, 7% chopped barley straw, and 40% concentrate. Corn silage was prepared in September 2023. Ensilage was performed in a bunker silo using a silage additive containing a mixture of *Lactobacillus buchneri* and *Lactobacillus plantarum* (Pioneer^®^ 11C33, Corteva Agriscience^TM^, Lisboa, Portugal). A common concentrate mixture was formulated with 15% of SH included in the control diet. Increasing dietary levels of DGP (3 and 6%, DM basis) were achieved by replacing half or all of SH with a commercial DGP (VINIFEED, which is a dehydrated and micronized grape pomace from wine production followed by distillation, composed of grape skins, pulp, and seeds, and approved for use in compound feed for all animal species; Destilaria Levira, Lda, Anadia, Portugal). The experimental diets were named according to their DGP content: 0% DGP (DGP0), 3% (DGP3), and 6% (DGP6; Table 1).

Diets were fed as TMR for ad libitum intake, with fresh feed offered twice each day (9:00 a.m. and 6:30 p.m.). Orts were collected and weighed daily after the morning milking. The amount of feed offered was adjusted each week to produce weigh-backs of approximately 5%. Samples of experimental feeds and orts were collected on three non-consecutive days during each measurement period for chemical composition analysis. Cows were milked twice daily at 8:00 a.m. and 6:00 p.m. Milk production was measured throughout the experimental period. Milk was sampled at both milkings on two consecutive days during the final day of each measurement period and analyzed for fat, protein, lactose, and milk urea N (MUN) by mid-infrared spectroscopy (Fossomatic^TM^ + MilkoScan^TM^ 7 RM, Foss Analytical, Hillerød, Denmark). Milk composition is reported from separate analyses adjusted proportionally based on milk yield. At the end of the experiment, cows continued to be fed the experimental diets and were subjected to the same management to determine the in situ degradability of SH and DGP.

### 2.2. Rumen Content Collections and In Situ Degradability

On the penultimate day of the experimental period, rumen contents were collected four hours after the morning feeding. The pH was measured immediately, and subsamples were frozen at −20 °C for later analysis. The nylon bag technique [16] was used to measure the N degradation of RSM and SBM in the rumen during the final 2 d of each experimental period. Nylon bags (10 × 20 cm; Bar Diamond, Inc., Parma, ID, USA) containing 5 g of each ground protein source (through a 4 mm screen) were incubated in the rumen of each cow in duplicate for 1, 2, 4, 8, 16, 24, and 48 h. Immediately after removal from the rumen, the bags were washed in cold water and frozen at −18 °C. At the end of the collections, the bags were thawed and washed in a washing machine for 40 min at 40 °C. Then, they were dried at 65 °C for 48 h. The dried residues were analyzed for Kjeldahl N. The same procedure was used to determine the in situ degradability of SH and DGP, except for the incubation times, which were 3, 9, 16, 24, 36, 48, 60, 72, 96, 120, 144, and 168 h. Dried residues of DGP from 9, 16, 24, and 48 h incubations from cows fed DGP0 and DGP3 diets were also submitted for condensed tannin analysis.

### 2.3. Chemical Analysis

Ground samples (1 mm) of experimental raw materials, corn silage, barley straw, and concentrate mixtures were analyzed in duplicate according to AOAC [17] for DM (method 934.01), ash (method 942.05), ether extract (method 920.39), and Kjeldahl N (method 954.01). Crude protein was calculated as Kjeldahl N × 6.25 (method 954.01). Neutral detergent fiber, acid detergent fiber (ADF), and acid detergent lignin (ADL) were determined by the detergent procedures of Van Soest, Robertson [18] and Robertson and Van Soest [19]. During NDF extraction, α-amylase was added, except for barley straw; sodium sulfite was not added. Neutral detergent fiber was expressed without residual ash. Neutral detergent insoluble N (NDIN) and acid detergent insoluble N (ADIN) were determined on NDF and ADF residues, respectively, using the Kjeldahl method. Starch was analyzed in ground samples (0.5 mm) using the method described by Salomonsson, Theander [20]. Phosphorus was determined by spectrophotometry, according to Commission Regulation (EC) No. 152/2009 Annex III, Part P [21], based on ISO 6491 [22]. Condensed tannins were quantified in DGP and DGP nylon bag residue samples by Creative Proteomics (Shirley, NY, USA), using a Thermo Scientific™ Multiskan™ GO instrument., Finland.

To determine the total short-chain fatty acids (SCFA) production and the individual SCFA profile in rumen fluid samples, an aliquot of 1.00 mL was transferred into a 2.0 mL Eppendorf tube and mixed with 250 µL of a 25% *w*/*w* H_3_PO_4_ solution for protein precipitation. The mixture was homogenized and subsequently centrifuged at 12,000 rpm for 20 min at 4 °C. Next, 250 µL of the supernatant was transferred into 1.5 mL-capacity GC vials containing 250 µL of ultrapure water and 500 µL of the internal standard solution (1.00 mmol/L, 3-methylvaleric acid). The final mixture was homogenized prior to GC-FID analysis. The quantification of the analytes was performed using the external calibration method. Five calibration standards were prepared, following the procedure referred to for the samples’ preparation, with the following modifications: instead of a 250 µL sample, 250 µL of a mixture 25% *w*/*w* H_3_PO_4_, water (1:4, *v*/*v*) were measured; and instead of 250 µL of ultrapure water, 250 µL of different standard solutions containing a mixture of volatile carboxylic acids were measured. The abovementioned volume and concentration of the internal standard solution were used in the preparation of the calibration standards. Coefficient correlations (r) > 0.999 were required for quantification purposes. A gas chromatography system GC-FID (Shimadzu GC-2010 Plus, Shimadzu Corporation, Kyoto, Japan) featuring a capillary column HP-FFAP (19091F-443), 30 m × 0.25 mm, 0.25 µm (Agilent Technologies, Santa Clara, CA, USA) was used. The chromatographic conditions were as follows: sample volume—1 µL, split injection ratio—1:50, injector temperature—260 °C, and FID temperature—260 °C. The temperature of the oven was as follows: 100 °C (5 min), with a temperature increase rate of 6 °C min^−1^ until 220 °C, and 220 °C (for 5 min). Dinitrogen was used as a carrier gas. The chromatographic run was 30 min. The retention times of the analytes were as follows: acetic acid, 8.8 min; propionic acid, 10.8 min; isobutyric acid, 11.4 min; butyric acid, 12.8 min; isovaleric acid, 13.7 min; valeric acid, 15.2 min; 3-methylvaleric acid, 16.4 min; isocaproic acid, 16.7 min; and caproic acid, 17.4 min.

### 2.4. Statistical Analysis

The experiment was designed, taking into consideration the recommendations of Vanzant, Cochran [23], which were recently updated by Foster, Smith [24] regarding the standardization of the in situ technique, namely ensuring a minimum number of 2 animals and 2 days. The 9.4 version of software package SAS^®^ OnDemand for Academics (SAS Institute Inc., Cary, NC, USA, 2025) was used for all statistical analyses.

Performance data and rumen function parameters were analyzed according to a 3 × 3 Latin square design, with the general linear model (PROC GLM) including the fixed effects of period, cow, and diet, and the residual error. Orthogonal polynomial contrasts were applied to test for linear and quadratic effects of diet. A significant effect was set when *p* < 0.05. Dry matter and N degradation data of DGP and SH were fitted using the nonlinear regression procedure (PROC NLIN) to the Ørskov and McDonald [25] model:

$$P\;=\;a\;+\;b\;\times \left(1\;-\;e^{-c\;\times \;t}\right)$$

where P is the degradation at time t, a is the soluble fraction, b is the insoluble but potentially degradable fraction, and c is the fractional degradation rate of b. The Marquardt iterative method was used to estimate the model parameters.

Nitrogen degradation data from protein sources were further analyzed using a nonlinear mixed-effects model with the PROC NLMIXED procedure. The kinetics of N degradation over time were described using the Ørskov and McDonald [25] model:

$$Y_{ijk}=\;a_{ij}+\;b_{ij}\left(1\;-\;e^{-c_{ijk}\;\times \;t_{k}}\right)+\;\beta_{p_{k}}+\;u_{i}+\;\varepsilon_{ijk}$$

where $Y_{ijk}$ is the N degradation for cow i, protein source j, and incubation time $t_{k}$; $a_{ij}$ is the soluble fraction; $b_{ij}$ is the potentially degradable fraction; $c_{ijk}$ is the fractional degradation rate; $\beta_{p_{k}}$ is the fixed effect of period k; $u_{i}$ is the random effect of cow i, with $u_{i}\sim N\left(0,\;\sigma_{Cow}^{2}\right)$; and $\varepsilon_{ijk}$ is the residual error, assuming $\varepsilon_{ijk}\;\sim \;N\left(0,\;\sigma^{2}\right)$. Parameters a and b were specified separately for each protein source (RSM or SBM), independent of diet. In contrast, the parameter c was specified for each combination of protein source (RSM or SBM) and diet (DGP0, DGP3, or DGP6). The model included random intercepts for individual cows to account for repeated measures, and residual variance was assumed to be constant across observations. Model parameters were estimated using the quasi-Newton (QUANEW) optimization algorithm. Convergence criteria and model diagnostics followed the default settings of the procedure. Random effects and residual variances were assumed to follow normal distributions as described above. Orthogonal contrasts were used to assess trends in c parameter across diets, separately for each protein source (RSM and SBM), and combined.

## 3. Results

Experimental diets differed only in the replacement of SH by DGP up to 6% (DM basis), presenting a similar chemical composition. Crude protein, NDF, and starch contents averaged ca. 15%, 37%, and 21% (DM basis), respectively (Table 1). Compared to SH, DGP presented slightly lower ash content and higher CP, EE, and fiber contents, and particularly high ADL content (56%, DM basis; Table 2). The condensed tannin content represented 8.9% of DGP. The portion of N bound to the NDF (NDIN) and ADF (ADIN) in DGP was 47.8% and 39.1% of the total CP content, respectively (Table 2). Among the protein sources studied, RSM presented lower CP and higher fiber contents than SBM (Table 2).

**Figure 1:**
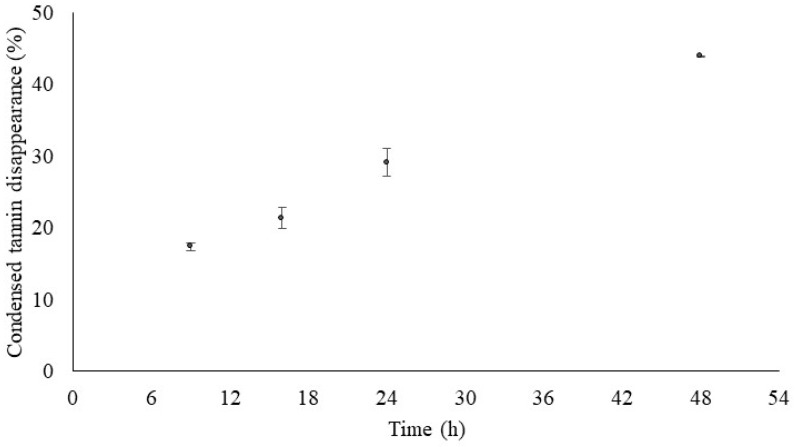
In situ condensed tannin disappearance from dehydrated grape pomace (%; mean ± SE).

The constants of the fitted exponential equations for ruminal DM and N degradation of DGP and SH are presented in Table 3. The model showed a good fit to the data (*p* < 0.001), with the highest root mean square error being 6.81. Regarding DGP, 41.7% of DM and 72.7% of N were potentially degradable (a+b), being the soluble (a), insoluble but potentially degradable $\left(b\right)$ fractions, and degradation rate (c), respectively, 27.3%, 14.4%, and 0.034 h^−1^ for DM, and 52.9%, 19.8%, and 0.039 h^−1^ for N. For SH, the percentage of DM and N potentially degradable was higher than 90%, the soluble fractions being lower (9.5% and 34.8% for DM and N, respectively) than those observed for DGP. In situ condensed tannin disappearance from DGP increased over time, reaching almost 50% after 48 h of incubation (Figure 1).

### 3.1. Feed Intake, Milk Production, and Milk Composition

Treatment effects on DM intake (DMI), milk production, milk composition, and feed efficiency are presented in Table 4. No diet effect was observed for DMI and milk production that averaged 27 kg/d and 37 kg/d, respectively. Similarly, the replacement of SH with DGP kept unaffected milk composition, except for MUN, for which a significant quadratic effect (*p* < 0.001) was observed. Feed efficiency, measured as milk production or ECM per DMI, was also not affected by diet.

### 3.2. Ruminal Fermentation Parameters

Table 5 presents the ruminal fermentation parameters from the different dietary treatments. Although rumen pH was not affected by the dietary treatment, dietary DGP inclusion quadratically reduced total ruminal SCFA concentration. Compared to the control, dietary inclusion of DGP reduced SCFA production by an average of 10%. The rumen SCFA profile was not affected by dietary treatments, except for a tendency toward a linear reduction in caproate proportion with DGP inclusion.

### 3.3. In Situ Degradability of Protein Sources

Regardless of the diet offered to cows, the potentially ruminal degradable fractions of N from RSM and SBM reached 93.3% and 99.6%, respectively (Table 6). The N degradation rate of RSM was not significantly affected by the dietary replacement of SH with DGP, whereas a tendency (*p* = 0.108) for a linear increase was observed for SBM (Table 6). A tendency for a linear increase in the N degradation rate was also observed when combining protein sources across diets (*p* = 0.101).

## 4. Discussion

The present study evaluated the effects of replacing SH with DGP in a common complementary compound feed formula for corn silage-based TMR on dairy cow performance, along with the impact of dietary DGP level on in situ rumen degradability of two common protein sources with known different degradation rates (RSM and SBM).

The chemical composition of GP is affected by grape variety, viticulture practices, type of winemaking process, and extraction techniques, among others [27,28]. Cell wall polysaccharides and lignin were the main constituents of the DGP studied, agreeing with earlier studies [29,30]. In addition, the low fiber digestibility and energy content of DGP contribute to its reduced use in diets for high-producing animals [30,31]. Grape pomace also presents a considerable content of tannins, polyphenolic compounds, and secondary metabolites of higher plants, whose effects depend on the chemical structure, dietary concentration, animal species involved, and composition of the basal diet [32,33,34]. Tannins are classified into two groups: hydrolysable tannins that yield water-soluble compounds (e.g., gallic acid, ellagic acid, pyrogallol, and sugars) through acid- or enzyme-catalyzed hydrolysis, and condensed tannins, a much larger group that is generally assumed to be indigestible [35,36]. However, we found that nearly half of the condensed tannins disappeared after 48 h of rumen incubation. Although in the present study, a characterization of tannins profile of DGP was not performed, an earlier study reported the existence of free, fiber-bound, and protein-bound tannins in GP, with the latter constituting the main fraction [29]. Additionally, when evaluating the rumen degradation of tropical tannin-rich plants, Rira, Morgavi [37] found that free condensed tannins from all plants completely disappeared after 24 h incubation in the rumen, whereas the disappearance of protein-bound condensed tannins greatly varied between plants (ranging from 93% for *Gliricidia sepium* to 21% for *Acacia nilótica*), and fibre-bound condensed tannin disappearance averaged 82% and did not vary between plants. Moreover, authors stated that the presence of tannins interferes with the microbial colonization of plants and that adherent communities in tannin-rich plants had a lower relative abundance of fibrolytic microbes, whereas archaea diversity was reduced in high tannin-containing plants. Although other studies also reported that rumen microorganisms degrade condensed tannins [38,39,40,41], further research is needed to understand how these microorganisms utilize them.

Although there were significant differences in energetic value and tannin content among the raw materials studied, directly replacing SH with DGP did not affect DMI in the present study. This outcome might be attributed to the low level of DGP inclusion (0.53% condensed tannins, DM basis, for DGP6), as a reduction in feed intake has been reported in ruminants consuming plants with high concentrations of condensed tannins (usually >5% DM), but not with moderate or low intake <5% DM [42,43]. Despite high amounts of condensed tannins impairing palatability and feed intake, some animals (e.g., deer) can adapt to tannin-rich diets by increasing the secretion of proline-rich proteins in the saliva. These proteins bind tannins, preventing their interaction with dietary protein [44]. However, domestic sheep produce these proteins only when consuming tannin-rich plants, while cattle do not show increased synthesis in response to tannin consumption [45]. This suggests that the effects of tannins are of greater importance in domestic cattle. No dietary effects were also observed for milk yield or milk solids content. The majority of the studies report no significant effects of DGP on milk production and composition among different dietary levels of DGP inclusion, e.g., 2% of DGP [46]. Chedea, Pelmus [47] also found no significant variations in the total amount of milk fat and protein of cows fed a diet supplemented with 15% DGP, but increased lactose concentration. Regarding protein profile, the authors observed an increase in β-lactoglobulin concentration, whereas there was no effect for α-lactalbumin, albumin, and caseins.

Milk urea N significantly increased quadratically with dietary DGP inclusion. This suggests that MUN was affected by a combination of two factors. First, certainly, there was an imbalance between the supply of fermentable energy and RDP [48,49], since the effective degradability of DM for a rumen outflow rate of 0.08 h^−1^ was 31.6% and 40.2% for DGP and SH, respectively; and the diets were not adjusted for the ratio of fermentable energy to RDP in order to avoid confounding effects. Second, the bioactive compounds in DGP may alter rumen function, which could partially explain why the effect was not linear. These agree with the observed effects of dietary DGP inclusion on total ruminal SCFA concentration and caproate molar proportion. Conversely, Nielsen and Hansen [50] reported a reduction in MUN content in dairy cows supplemented with 4.5 g/cow/d of a commercial additive containing GP extracts. Ream, Stevens [51] observed a reduction in urinary excretion of N and urea-N in beef cattle fed diets containing 15% DGP (DM basis). However, Vinyard, Myers [52] found quadratic effects on fecal N excretion, fecal fiber-bound N excretion, microbial N flow, and apparent N retention in crossbred beef heifers fed diets containing 0%, 15%, or 30% DGP (DM basis), but no effect on urinary excretion of N and urea-N.

Most studies report lower N degradation rates in the rumen when animals are fed diets containing tannins, particularly DGP, due to their ability to form protein complexes [53,54]. Additionally, adding tannin-rich feeds to diets has been shown to affect the abundance of different microbial genera. However, the results are inconsistent across studies, especially concerning cellulolytic and methanogenic microorganisms [55,56,57,58]. Conversely, the present study found that the N degradation rate of RSM was unaffected by replacing SH with DGP. However, a tendency for a linear increase was observed for SBM, which was also detected when combining protein sources across diets.

To the best of our knowledge, no previous studies have reported the use of an in situ technique to evaluate protein sources’ rumen degradation or the rumen fiber degradation of forages in cows fed diets with different amounts of DGP. This makes it difficult to compare results. One possible explanation for the findings is that moderate DGP supplementation was used, which may have selected microbial populations capable of tolerating or metabolizing polyphenols and tannins. Indeed, Biscarini, Palazzo [59] demonstrated in calves that red GP supplementation (10%, DM basis) enriched genera such as *Ruminiclostridium* and *Eubacterium*, whose functions are associated with flavonoid and xyloglucan degradation. Recently, Akter, Li [14] reported that supplementing up to 15% GP (DM basis) reduced CH_4_ emissions and suggested that the phenolic compounds and organic acids present in GP might be partially responsible for the observed reduction.

Despite the fact that data on community structure or functional genes are not provided herein, findings of the present study suggest that moderate DGP supplementation may affect rumen function, namely, promoting shifts in microbial adaptation, community composition, and fermentative dynamics. Moreover, the conflicting results observed in the literature regarding the effects of the dietary inclusion of DGP and other tannin-rich feeds require a more complete understanding of what is occurring within the rumen ecosystem at both the microbial and functional levels. In this context, follow-up studies employing omic tools (e.g., metagenomics, metatranscriptomics, and metabolomics), combined with detailed microbiota profiling, are crucial for clarifying whether the observed effects are primarily mediated by shifts in microbial composition or by the modulation of microbial activity by polyphenols, or by both processes in synergy.

## 5. Conclusions

The current study demonstrates that including up to 6% DGP in place of SH in corn silage-based diets has no detrimental effects on the feed intake, milk production, or composition of high-producing dairy cows, but quadratically increases MUN and reduces ruminal concentration of SCFA. An increasing tendency in the N degradation rate of SBM was observed with an increase in dietary DGP inclusion, which was also detected when combining protein sources across diets. The results underscore the importance of balancing the dietary supply of fermentable energy and RDP when replacing fiber sources with DGP. They also highlight the need for integrative studies combining animal performance with microbial, biochemical, and omics-based analyses to achieve a more comprehensive understanding of the effects. Valorizing DGP as a feed resource for high-producing animals from a circular economy perspective contributes to the sustainability of the dairy sector and mitigates the environmental damage caused by its disposal.

## Figures and Tables

**Table 1:** Ingredients and chemical composition of the experimental diets.

		Diet ^1^	
Item	DGP0	DGP3	DGP6
Ingredient (% of DM)			
Corn silage	53	53	53
Chopped barley straw	7.0	7.0	7.0
Corn grain	7.8	7.8	7.8
Rapeseed meal	12	12	12
Soybean meal	4.0	4.0	4.0
Sunflower meal	6.0	6.0	6.0
Soybean hulls	6.0	3.0	-
Dehydrated grape pomace	-	3.0	6.0
Cane molasses	1.2	1.2	1.2
Calcium carbonate	0.84	0.84	0.84
Calcium soaps ^2^	0.80	0.80	0.80
Urea	0.40	0.40	0.40
Sodium bicarbonate	0.36	0.36	0.36
Magnesium oxide	0.24	0.24	0.24
Mineral and vitamin premix ^3^	0.16	0.16	0.16
Salt	0.20	0.20	0.20
Chemical composition (% of DM, except for DM content)			
DM (%)	60.6	60.6	60.6
Ash	6.35	6.38	6.31
CP	15.1	15.1	15.1
EE	3.51	3.49	3.52
NDF	36.8	37.0	36.8
ADF	22.5	22.7	22.8
ADL	5.4	6.1	6.7
Starch	21.6	21.3	21.5
P	0.38	0.38	0.37

^1^ Diets are named according to their dehydrated grape pomace (DGP) content: DGP0 = 0%, DGP3 = 3%, and DGP6 = 6%. ^2^ Calcium salts of palm fatty acid distillate, SOLAFAM 424, AFAMSA, Pontevedra, Spain. ^3^ Contained: 5,000,000 IU/kg of vitamin A; 750,000 IU/kg of vitamin D_3_; 20,000 mg/kg of vitamin E; 375 mg/kg of vitamin B_6_; 1313 mg/kg of vitamin C; 500 mg/kg of beta-carotene; 13,310 mg/kg of betaine anhydrous; 2500 mg/kg of Fe; 17,500 mg/kg of Zn; 5000 mg/kg of Cu; 15,000 mg/kg of Mn; 450 mg/kg of I; 98 mg/kg of Co; 15 mg/kg of Se; and 75 mg/kg of organic Se.

**Table 2:** Chemical composition of experimental raw materials (% of DM, unless otherwise indicated) ^1^.

Item	DGP	SH	RSM	SBM
DM (%)	90.6	90.8	90.9	89.4
Ash	5.27	7.90	5.24	7.41
CP	11.5	9.45	39.4	49.1
EE	3.58	1.03	1.61	1.46
NDF	73.4	70.5	28.8	18.9
ADF	65.3	51.2	21.2	10.7
ADL	56.2	5.83	11.7	4.22
NDIN (% of CP)	47.8	ND ^2^	ND	ND
ADIN (% of CP)	39.1	ND	ND	ND
P	0.26	0.07	1.26	0.66
Condensed tannins	8.90	ND	ND	ND

^1^ DGP = dehydrated grape pomace; SH = soybean hulls; RSM = rapeseed meal SMB = soybean meal. ^2^ ND = Not determined.

**Table 3:** Rumen degradation parameters of tested dehydrated grape pomace (DGP) and soybean hulls (SH).

	a	SE	b	SE	c	SE	(a+b)	RMSE ^1^	*p*
DM									
DGP	27.3	1.50	14.4	1.47	0.034	0.0085	41.7	2.48	<0.001
SH	9.5	4.73	83.0	4.59	0.047	0.0056	92.5	6.81	<0.001
N									
DGP	52.9	1.59	19.8	1.55	0.039	0.0070	72.7	2.50	<0.001
SH	34.8	2.96	59.2	2.88	0.055	0.0055	93.9	3.92	<0.001

^1^ Root mean square error.

**Table 4:** Least square means for feed intake, milk production and composition, and feed efficiency from the different dietary treatments.

		Diet ^1^			Contrasts (*p*) ^2^	
Item	DGP0	DGP3	DGP6	SEM	L	Q
DMI (kg/d)	27.2	26.9	27.3	0.81	0.938	0.765
Yield (kg/d)						
Milk	37.4	38.0	36.9	0.46	0.559	0.282
ECM ^3^	37.0	38.0	37.5	0.79	0.707	0.505
Fat	1.48	1.53	1.53	0.074	0.670	0.821
Protein	1.18	1.22	1.17	0.017	0.933	0.204
Lactose	1.83	1.85	1.80	0.030	0.578	0.418
Composition (%)						
Fat	3.97	4.05	4.20	0.226	0.552	0.902
Protein	3.14	3.21	3.20	0.057	0.564	0.650
Lactose	4.89	4.87	4.87	0.018	0.545	0.646
MUN (mg/d)	14.5	16.5	17.3	0.01	<0.001	<0.001
Milk/DMI	1.38	1.42	1.35	0.036	0.632	0.343
ECM/DMI	1.37	1.42	1.37	0.068	0.943	0.592

^1^ Diets are named according to their dehydrated grape pomace (DGP) content: DGP0 = 0%, DGP3 = 3%, and DGP6 = 6%. ^2^ L = linear and Q = quadratic. ^3^ ECM = 12.51 × kg Fat + 7.41 × kg CP + 5.32 × kg Lactose [26].

**Table 5:** Least square means for ruminal fermentation parameters from the different dietary treatments.

		Diet ^1^			Contrasts (*p*) ^2^	
Item	DGP0	DGP3	DGP6	SEM	L	Q
pH	6.0	5.9	6.1	0.18	0.805	0.604
Total SCFA ^3^ (mmol/L)	151.3	134.0	138.1	1.84	0.037	0.042
SCFA profile (% mol)						
Acetate	62.1	63.9	63.4	1.03	0.477	0.455
Propionate	19.7	18.0	19.6	0.95	0.927	0.288
Isobutyrate	0.89	0.85	0.95	0.052	0.504	0.413
Butyrate	13.5	13.1	12.2	0.41	0.154	0.669
Isovarelate	1.82	2.34	2.10	0.278	0.548	0.385
Valerate	1.45	1.36	1.39	0.056	0.569	0.488
Caproate	0.50	0.45	0.41	0.018	0.064	0.972
Acetate/Propionate	3.18	3.58	3.29	0.211	0.750	0.321
Propionate/Butyrate	1.46	1.37	1.68	0.140	0.389	0.369

^1^ Diets are named according to their dehydrated grape pomace (DGP) content: DGP0 = 0%, DGP3 = 3%, and DGP6 = 6%. ^2^ L = linear and Q = quadratic. ^3^ SCFA = short-chain fatty acids.

**Table 6:** Nitrogen degradation parameters of tested rapeseed meal (RSM) and soybean meal (SBM) ^1,2,3^.

	a	SE	b	SE	cDGP0	SE	cDGP3	SE	cDGP6	SE	L ^4^	Q ^4^
RSM	31.9	1.38	61.3	1.66	0.095	0.0089	0.103	0.0105	0.111	0.0107	0.234	0.998
SBM	18.8	1.30	80.8	1.71	0.076	0.0055	0.087	0.0063	0.093	0.0070	0.108	0.752

^1^ Parameters a and b were specified separately for each protein source, independent of diet, and, conversely, the parameter c was specified for each combination of protein source and diet (named according to their dehydrated grape pomace (DGP) content: DGP0 = 0%, DGP3 = 3%, and DGP6 = 6%). ^2^ Root mean square error (RMSE) = 4.33, calculated using the predicted values from the model and the observed data. ^3^ *p*-values of orthogonal contrasts combining protein sources for the c parameters across diets, for linear and quadratic effects, were 0.101 and 0.870, respectively. ^4^ *p*-values of linear and quadratic orthogonal contrasts for the c parameter across diets within protein sources.

## Data Availability

Restrictions apply to the datasets: The datasets presented in this article are not readily available because the data are part of an ongoing project. Requests to access the datasets should be directed to the corresponding authors.
